# The role of carotid elongation for intervention time and outcome in mechanical thrombectomy for anterior circulation acute ischemic stroke

**DOI:** 10.1007/s00234-024-03539-0

**Published:** 2025-01-08

**Authors:** Vivien Lorena Ivan, Christian Rubbert, Daniel Weiß, Luisa Wolf, Marius Vach, Marius Kaschner, Bernd Turowski, Michael Gliem, John-Ih Lee, Tobias Ruck, Julian Caspers

**Affiliations:** 1 Department of Diagnostic and Interventional Radiology, Medical Faculty and University Hospital Düsseldorf, Heinrich-Heine-University, Düsseldorf, Germany; 2 Department of Neurology, Medical Faculty and University Hospital Düsseldorf, Heinrich-Heine-University, Düsseldorf, Germany

**Keywords:** Carotid arteries, Stroke, Thrombectomy, Computed tomography angiography

## Abstract

**Introduction:**

This study investigates the influence of carotid artery elongation on neurovascular intervention and outcome in acute stroke treatments proposing an easily assessable imaging marker for carotid elongation.

**Methods:**

118 patients who underwent mechanical thrombectomy for middle cerebral artery occlusions were included. The carotid elongation ratio (CER), center-line artery length to scan’s Z-axis, was measured on the affected side in CT-angiographies. Full and partial correlations of CER with periprocedural times, complications and outcome were computed. Multivariate logistic regression, including comorbidities, for prediction of dichotomized mRS outcome after 3 months was performed.

**Results:**

CER showed no significant correlation with recanalization success. Weak, outlier-driven correlation was found with recanalization time (*p* = 0.021, cor = 0.2). Weak correlations were found with improvement of NIHSS score at discharge and mRS score after 3 months (*p* = 0.023 and *p* = 0.031, each rho=-0.2). There was moderate correlation with NIHSS score at discharge (*p* = 0.001, rho = 0.3). Patients with favorable outcomes (mRS 0–2) exhibited lower CER (*p* = 0.012). Partial correlations of CER with favorable outcomes were observed after correcting for age, sex and cardiovascular risk factors (cor = 0.2, *p* = 0.048). Multivariate analysis (Nagelkerke’s R2 = 0.42) identified NIHSS score at admission, diabetes, hypertension and intervention time as significant factors for predicting outcome at 3 month, while CER showed the highest log Odd’s (2.97).

**Conclusion:**

Correlations between CER and clinical improvement suggest that carotid elongation might be a risk factor for poorer outcome without relevant effect on endovascular treatment and should not guide treatment decisions. Further studies should consider carotid elongation as an individual neurovascular risk factor, independent of hypertension.

## Key points

•The study investigates the influence of carotid elongation on endovascular stroke treatment and outcome.

•There is correlation between carotid elongation and clinical improvement. However, no relevant effect on endovascular treatment was found.

•Carotid elongation should not dictate acute stroke treatment. Rather it should be considered as an individual neurovascular risk factor.

## Introduction

Endovascular mechanical recanalization of large vessel occlusions has become an established procedure for treatment of acute ischemic stroke, particularly for middles cerebral artery and internal carotid artery occlusions, following the significant findings of the five major randomized controlled thrombectomy trials from 2015 (MR CLEAN, EXTEND IA, ESCAPE, REVASCAT, SWIFT PRIME) [1–5]. These studies clearly verified the efficacy and safety of this procedure and led to its inclusion in international treatment guidelines. However, the functional outcome of patients suffering from acute ischemic stroke is strongly dependent on time to recanalization [6]. Thus, time to recanalization and infrastructural improvements on procedural times have garnered significant attention in acute stroke care [6, 7]. 

Stroke patients typically have a higher cardiovascular risk profile. One manifestation of increased cardiovascular risk factors, particularly of arterial hypertension, is alteration in vascular structure with an increased tortuosity level [8, 9]. Mechanical thrombectomy requires extensive practice and expertise. However, the complexity of vascular anatomy in some patients poses an additional challenge. The access route for endovascular treatments targeting the anterior stroke pathway primarily involves navigating through the common carotid artery and the extra- and intracranial internal carotid artery. The presence of coils and kinks adds to the anatomical complexity. Collective experiences of neurointerventionalists and findings from various studies emphasize that a challenging anatomical access route due to vascular elongation represents a major reason for failed or prolonged endovascular recanalization and reduces the efficacy of mechanical thrombectomy [10, 11]. However, there is a need for further investigation to understand the effect of carotid elongation on procedural duration and patient outcomes.

We hypothesize that carotid elongation significantly influences the duration of intervention and impacts patient’s outcome in mechanical thrombectomy, potentially leading to poorer outcomes. Therefore, an easily assessable index for quantification of carotid artery elongation is investigated and analyzed for relations to procedural time of intervention and effects on patient outcomes in a sample of patients undergoing mechanical thrombectomy in anterior circulation stroke. We aim to assess a practical measure, largely independent of patient positioning, that effectively assesses the anatomical complexity in patients undergoing mechanical thrombectomy making it applicable in daily clinical use.

## Materials and methods

### Study design and data collection

In this work, we analyzed data from a prospective, single-center registry study that investigates the influence of imaging and clinical parameters on functional outcomes in patients undergoing mechanical thrombectomy for acute ischemic stroke [12–15].

The study was approved by the local ethics committee.

**Fig. 1:**
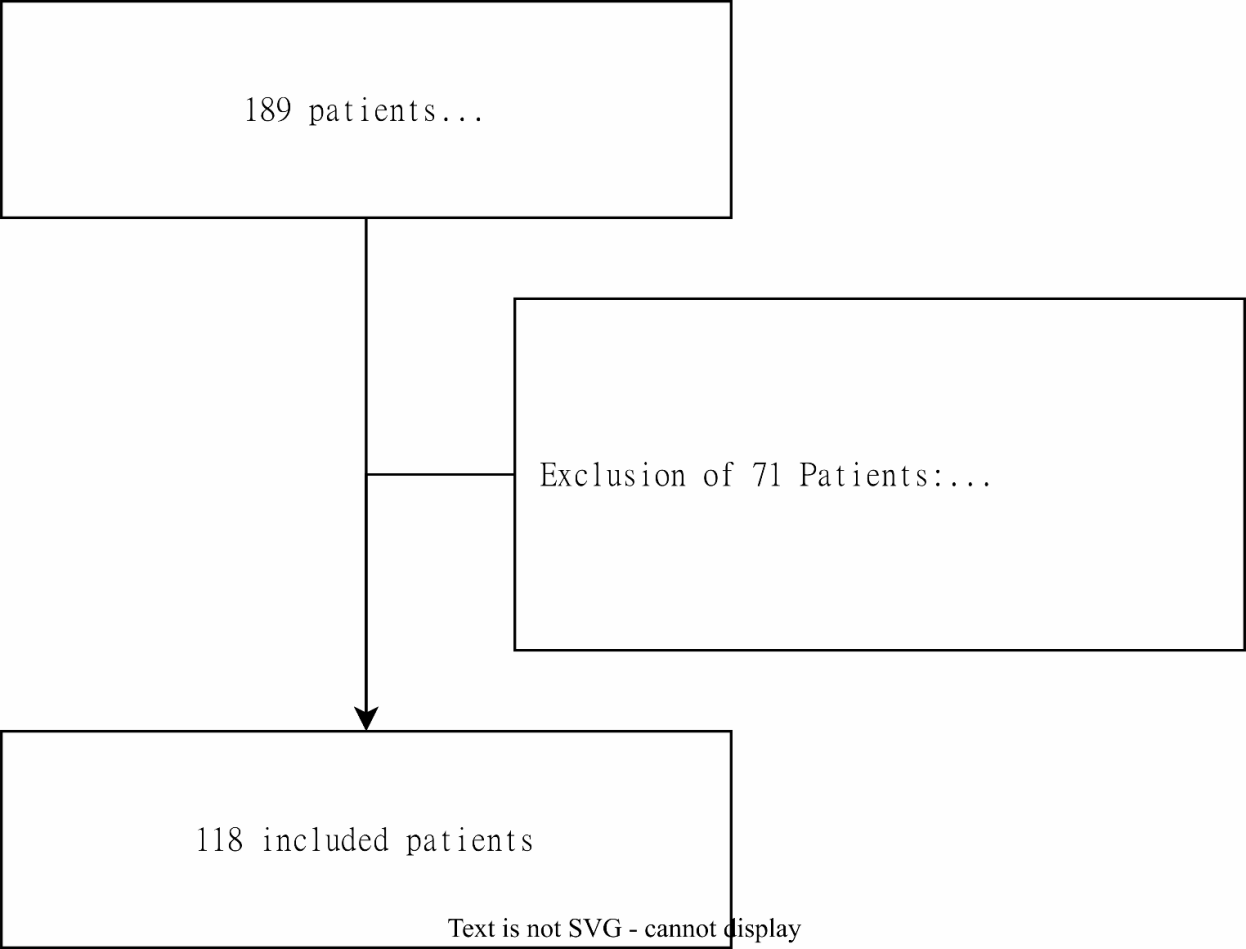
Study Selection Flowchart

The registry includes cases of mechanical thrombectomy for middle cerebral artery occlusions at our institution between 2016 and 2018, with available outcome data and pre-interventional imaging of the cervical and intracranial arteries. These cases were manually reviewed and screened according to the following exclusion criteria: (1) additional carotid artery occlusion on the affected side, (2) additional vascular dissection on the affected side, (3) initial performance of a Magnetic Resonance Imaging (MRI) instead of CTA, (4) incomplete assessment of the aortic arch on CTA and (5) no immediate endovascular intervention (Fig. 1).

Mechanical thrombectomy was conducted using Stent-Retriever thrombectomy in combination with aspiration catheters. Choice of materials was up to the interventionalist but followed a standard scheme: The local standard for anterior circulation stroke during study period included access via an 80 cm 8 French (F) sheath (Arrow-Flex^®^, Teleflex, Athlone, Ireland), together with or without an additional 8 F guiding catheter (8 F MPD Vista Brite Tip^®^, Cordis Corp., Hialeah, FL, USA), a 6 F aspiration catheter (SOFIA™, MicroVention Inc., Aliso Viejo, CA, USA), 0.021 inch microcatheter (NeuroSlider^®^, Acandis GmbH, Pforzheim, Germany) and 0.014 inch microwire (Synchro^®^, Stryker corp., Kalamazoo, MI, USA). Thrombectomy was usually performed with Aperio^®^ stent-retrievers (Acandis GmbH, Pforzheim, Germany) using the SAVE technique [16]. In some cases, thrombectomy was performed in SOLUMBRA technique or sole aspiration [17]. Only patients who underwent at least one thrombectomy maneuver or at least one aspiration were included in the study. Patients undergoing either planned stenting or emergently indicated stenting, such as for dissection, were explicitly excluded from the study.

To comprehensively characterize the sample, the following data were collected: presence of any of the previously mentioned exclusion criteria, sex, age, administration of intravenous thrombolysis, the affected hemisphere, and the presence of comorbidities, including diabetes, hypertension, atrial fibrillation, and peripheral arterial vascular disease (pAVD), as well as information on smoking habits.

Procedural data were recorded including specific time points: symptom onset time, intravenous thrombolysis time (if performed), groin puncture time, recanalization time. The following time intervals were calculated: recanalization interval (from groin puncture to successful recanalization), onset to groin puncture interval (from symptom onset to the start of thrombectomy) and onset to recanalization interval (from symptom onset to successful recanalization). Success of recanalization was assessed using the expanded and modified Thrombolysis In Cerebral Infarction (mTICI) scale including mTICI 0, mTICI 1, mTICI 2a, mTICI 2b, mTICI 2c and mTICI 3.

Patient outcomes were assessed using the National Institute of Health Stroke Scale (NIHSS) and the modified Rankin scale (mRS), both at admission and discharge. The mRS was additionally assessed after three months to evaluate follow up outcomes.

Hemorrhagic transformations and complications were collected and categorized in bleeding complications, cerebral edema, groin bleeding, femoral vessel occlusion and arterial dissection. Bleeding complications were classified according to the Heidelberg Bleeding Classification and ECASS Classification (European Cooperative Acute Stroke Study), which is integrated in the Heidelberg classification: class 1a: scattered small petechiae (HI1); class 1b: confluent petechiae (HI2); class 1c: hematoma within infarcted tissue, occupying < 30% (PH1), with no substantive mass effect; class 2: intracerebral hemorrhage within and beyond infarcted brain tissue (PH2); class 3a: parenchymal hematoma remote from infarcted brain tissue; class 3b: intraventricular hemorrhage; class 3c: subarachnoid hemorrhage; class 3d: subdural hemorrhage [18]. 

### Measurement of carotid elongation

**Fig. 2:**
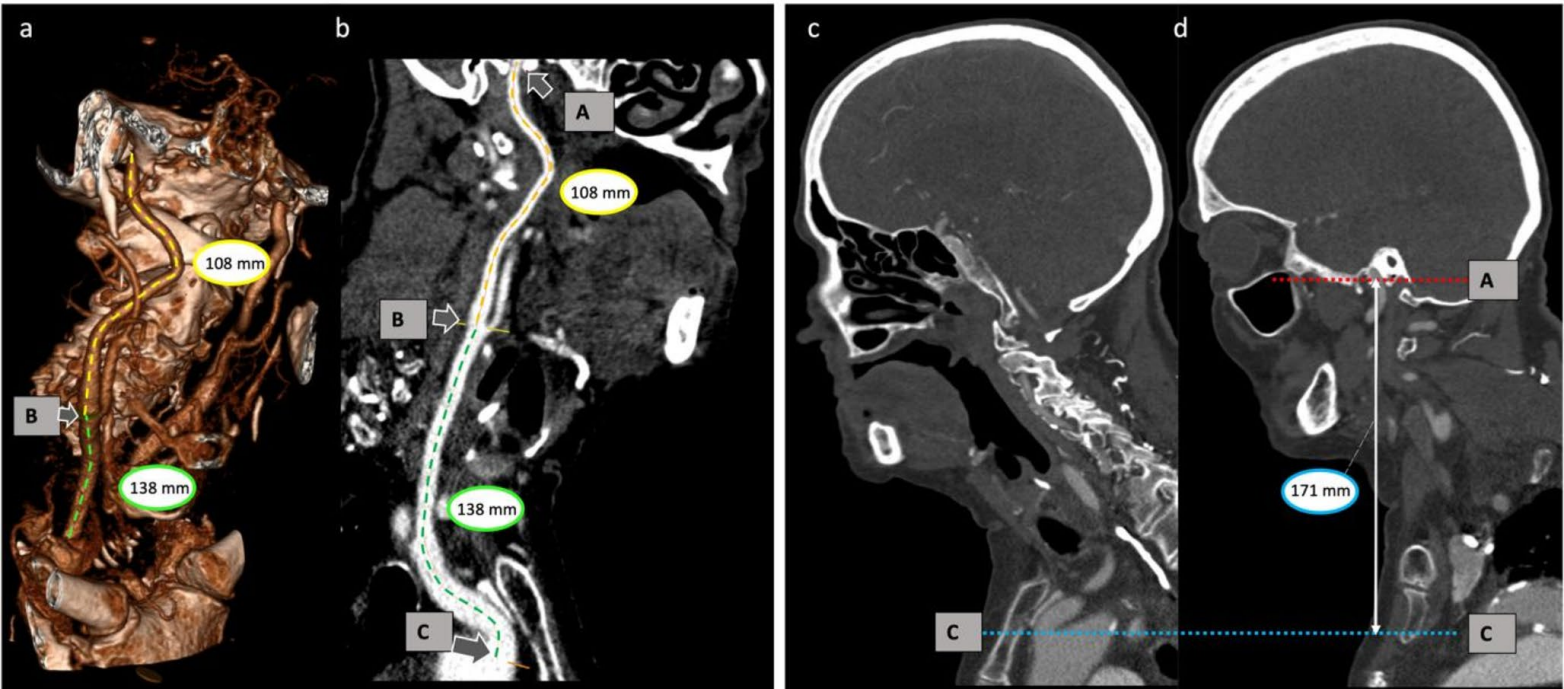
Measurement of Carotid Elongation Ratio. **a**. & **b**.: Carotid artery centerline measurement in 3D reconstruction (**a**) and vascular analysis view (**b**) using the 3D vessel analysis tool by Sectra AB [Sectra] IDS7/dx version 24.1.13.5511 (x64). Length of common carotid artery (green, 138 mm) and extracranial internal carotid artery (yellow, 108 mm). The total length of the carotid artery (length of the common carotid artery plus the extracranial internal carotid artery) is 108 mm + 138 mm = 246 mm. c. & d.: Z-axis measurement in lateral computed tomography angiography. Blue marker indicates the access artery’s origin from the aortic arch. Red marker indicates the entry point of the internal carotid artery into the skull base. The white line represents the Z-axis between these markers, measured parallel to the CT table. In this case, the Z-axis measures 171 mm. **a**. - **d**.: CER from this example: (138 + 108): 171 = 1.43. A: entry point of internal carotid artery into the skull base; B: bifurcation; C: artery’s origin from the aortic arch

Carotid artery measurements were performed on the side of the hemisphere affected by middle cerebral artery occlusion. To assess carotid elongation and tortuosity, we proposed an easy-to-assess imaging marker calculated as the ratio between the (centerline) extracranial carotid length (common and extracranial internal carotid artery) and the z-axis length of the scan (carotid elongation ratio; CER). Measurements were performed using the 3D vessel analysis tool implemented in the Sectra IDS7 Picture Archiving and Communication System (PACS) (IDS7/dx version 24.1.13.5511 (x64), Sectra AB, Linköping, Sweden). The length of the extracranial carotid artery (common and extracranial internal carotid artery) was measured from its arterial origin at the aortic arch to the entrance into the carotid canal of the petrous bone at the skull base along the arterial centerline. For the right side, the brachiocephalic trunk was included to the measurement of the carotid artery. In cases where the right carotid artery directly originates from the aortic arch, measurement started from the level of the aortic arch. The measurement was conducted for the entire extracranial carotid artery as described above and, additionally, for the common carotid artery and extracranial internal carotid artery separately. The transition point between common carotid artery and internal carotid artery was positioned at the level of the bifurcation (Fig. 2). The denominator of CER was defined as the Chebyshev distance [19] along the z-axis in the 3-dimensional room, which represents the straight distance parallel to the table and orthogonal to the scanning direction between both measurement points (Fig. 2). Even though the Euclidean distance could also serve as a viable option for this measurement, we chose a table-oriented approach in our methodology rather than a patient-oriented reference in order to enhance the simplicity of assessment without the need for individualized multiplanar reconstructions and, consequently, to improve the reproducibility of the measure.

Carotid artery measurements were performed on the side of the hemisphere affected by middle cerebral artery occlusion. To assess carotid elongation and tortuosity, we proposed an easy-to-assess imaging marker calculated as the ratio between the (centerline) extracranial carotid length (common and extracranial internal carotid artery) and the z-axis length of the scan (carotid elongation ratio; CER). Measurements were performed using the 3D vessel analysis tool implemented in the Sectra IDS7 Picture Archiving and Communication System (PACS) (IDS7/dx version 24.1.13.5511 (x64), Sectra AB, Linköping, Sweden). The length of the extracranial carotid artery (common and extracranial internal carotid artery) was measured from its arterial origin at the aortic arch to the entrance into the carotid canal of the petrous bone at the skull base along the arterial centerline. For the right side, the brachiocephalic trunk was included to the measurement of the carotid artery. In cases where the right carotid artery directly originates from the aortic arch, measurement started from the level of the aortic arch. The measurement was conducted for the entire extracranial carotid artery as described above and, additionally, for the common carotid artery and extracranial internal carotid artery separately. The transition point between common carotid artery and internal carotid artery was positioned at the level of the bifurcation (Fig. 2). The denominator of CER was defined as the Chebyshev distance [19] along the z-axis in the 3-dimensional room, which represents the straight distance parallel to the table and orthogonal to the scanning direction between both measurement points (Fig. 2). Even though the Euclidean distance could also serve as a viable option for this measurement, we chose a table-oriented approach in our methodology rather than a patient-oriented reference in order to enhance the simplicity of assessment without the need for individualized multiplanar reconstructions and, consequently, to improve the reproducibility of the measure.

The carotid elongation ratio was assessed by one author (VI, 3 years of experience), and was verified by another author reviewing all measurements (JC, 11 years of experience). Review focused on ensuring that the measurements were performed in accordance with the definition provided above and executed accurately to the defined measurement points. The raters were blinded to the clinical information.

### Statistical analysis

Statistical analysis was conducted using R statistics (R Core Team, version 4.3.0) [20]. Descriptive statistics were employed to summarize the demographic characteristics and procedural variables. The relationship between CER and the duration of intervention were analyzed using Pearson correlation analyses. Patients in which no reperfusion could be achieved in the endovascular procedure (i.e., mTICI 0) were not considered in the analysis of intervention time. The relationship between CER and patient outcomes as well as recanalization results, (post-) procedural complications and NIHSS at admission, were analyzed with correlation analyses. Additionally, partial correlation was performed between CER and dichotomized outcome controlling for age, sex, diabetes, arterial hypertension, and smoking habits. Group comparisons were performed by using unpaired two-sided Student’s t-test. For group comparisons, recanalization results as measured by the mTICI scale were dichotomized. Successful recanalization was defined as mTICI 2b-3 and poor recanalization as mTICI 0-2a. In a second analysis, a more conservative dichotomization threshold was used, i.e., by considering only mTICI 2c and mTICI 3 as successful recanalization. Another group comparison was performed examining CER between patients with favorable functional outcome (defined as mRS 0–2) and poor outcome (defined as mRS 3–6) at 3 months post-intervention. A binary logistic regression analysis including age, sex, CER, diabetes, arterial hypertension, atrial fibrillation, pAVD, smoking habits, NIHSS at admission, mTICI and intervention duration as features was conducted, considering Nagelkerke’s R^2^ and testing for multicollinearity.

A significance level of *p* < 0.05 was used to determine statistical significance for all statistical tests. A correlation level of rho / cor ≤ 0.2 was used to determine a weak correlation, rho / cor > 0.2 and ≤ 0.4 as moderate correlation and rho / cor > 0.4 was used to determine a strong correlation.

## Results

### Sample characteristics

In total, 189 patients undergoing endovascular treatment for acute ischemic stroke due to middle cerebral artery occlusion within the study interval were enrolled. A total of 118 patients (62.4%) were included in the final analysis. However, 71 patients (37.6%) were excluded from the study: 33 patients (17.5%) had an additional carotid artery occlusion on the affected side, two patients (1.1%) had an additional vascular dissection on the affected side, 26 patients (13.8%) underwent initial imaging with Magnetic Resonance Imaging (MRI) instead of CTA, 9 subjects (4.8%) showed incomplete assessment of the aortic arch on CTA, and one patient (0.6%) had no immediate endovascular intervention. Detailed distribution of sample characteristics is shown in Table 1.

**Table 1:** Sample characteristics

Characteristics	Patients
Included patients	*n* = 118
Sex	
female	*n* = 72 (61%)
male	*n* = 46 (39%)
Age	
median	75
range	45–94
IVT performed	*n* = 79 (67%)
Affected hemisphere	
left	*n* = 70 (59%)
right	*n* = 48 (41%)
Comorbidities	
diabetes	*n* = 37 (31%)
arterial hypertension	*n* = 107 (91%)
atrial fibrillation	*n* = 76 (64%)
pAVD	*n* = 9 (8%)
cigarette smoking	*n* = 92 (78%)
mTICI	
mTICI 3	*n* = 43 (36.4%)
mTICI 2c	*n* = 23 (19.5%)
mTICI 2b	*n* = 40 (33.9%)
mTICI 2a	*n* = 6 (5%)
mTICI 1	*n* = 0 (0%)
mTICI 0	*n* = 4 (3.4%)

mTICI modified Thrombolysis in cerebral infarction scale. IVT intravenous thrombolysis. pAVD peripheral arterial vascular disease

Carotid Elongation, Procedural Times, and Outcomes.

Carotid elongation ratio (CER) could be assessed in all included patients of the final analysis. CER ranged from 1.01 to 1.68 within the study population, with a mean of 1.29 ± 0.15 (see Table 2).

**Table 2:** Carotid artery assessments

	Common Carotid Artery (CCA) length [mm]	Extracranial Internal Carotid Artery (eICA) length [mm]	Entire Extracranial Carotid artery (eCA) length [mm]	CT Angiography scan Z-Axis [mm]	Carotid Elongation Ratio (CER)
Mean ± SD	137.0 ± 20.0	96.5 ± 14.4	233.6 ± 24.3	182.0 ± 18.5	**1.29 ± 0.15**
Median	130.5	94	229	181	**1.26**
Minimum	70	70	140	138	**1.01**
Maximum	188	141	295	241	**1.68**

CER carotid elongation ratio. SD standard deviation

**Fig. 3:**
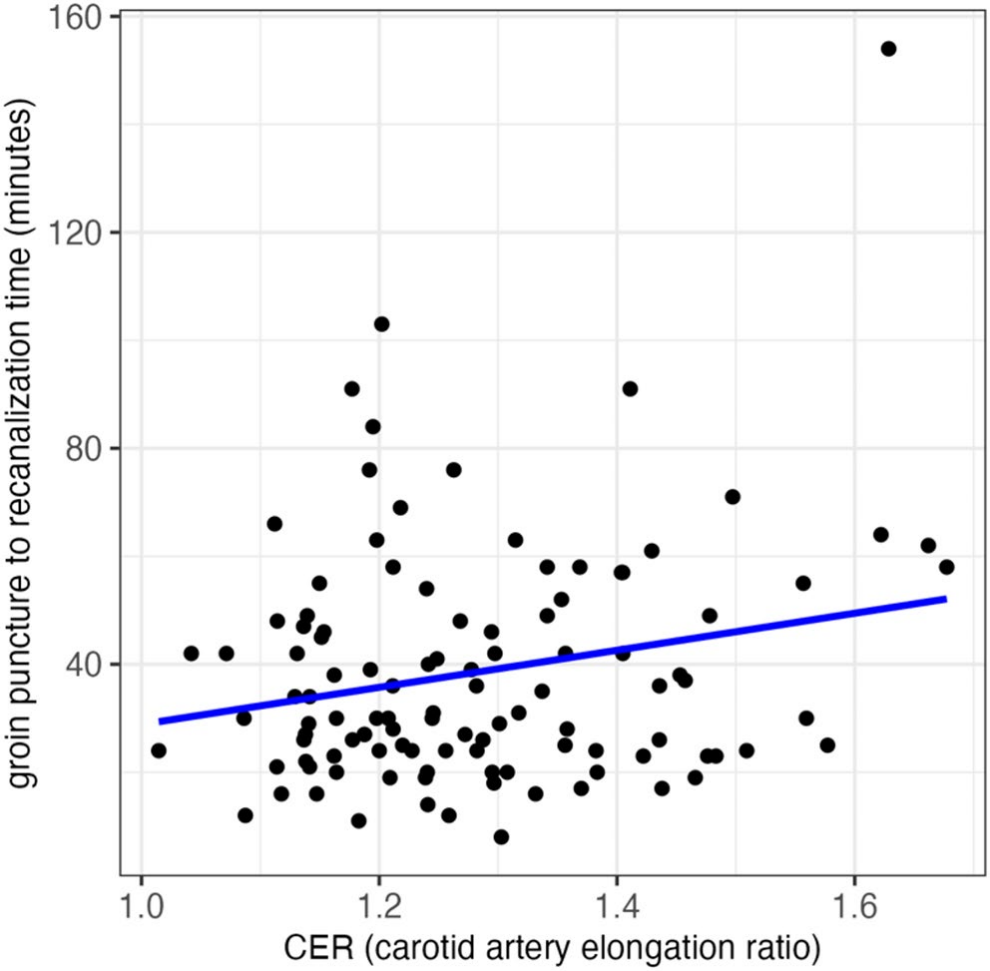
Correlation of groin puncture to recanalization time as a function of the CER (Carotid Elongation Ratio)

Mean time from stroke onset to groin puncture was 193 ± 79 min with a range from 50 to 575 min. Mean time from stroke onset to recanalization was 231 ± 79 min with a range from 67 to 611 min. The mean duration of the endovascular procedure, i.e., time from groin puncture to recanalization, was 39 ± 22 min with a range from 8 to 154 min. A statistically significant weak positive correlation between CER and recanalization time was found (*p* = 0.021, cor = 0.2; Fig. 3). The corresponding Fig. 3 reveals an outlier with a large CER and high intervention time. Reviewing the case, the patient exhibited vastly elongated vessels, resulting in a strongly prolonged intervention time. No co-occuring events or conditions were found that would warrant the exclusion of this data point, leading to its retention in the analysis. However, it should be noted that the exclusion of this outlier would have resulted in the absence of a statistically significant correlation between CER and recanalization time.

There was no significant correlation between the CER and recanalization success, as measured by the mTICI scale (*p* = 0.08). No significant group differences in CER were found between patients with successful recanalization, defined as mTICI 2b-3, and poor recanalization, defined as mTICI 0-2a (*p* = 0.37). Even with a more conservative dichotomization threshold, i.e., by considering only mTICI 2c and mTICI 3 as successful recanalization, no significant group differences between both recanalization groups could be observed (*p* = 0.07).

**Fig. 4:**
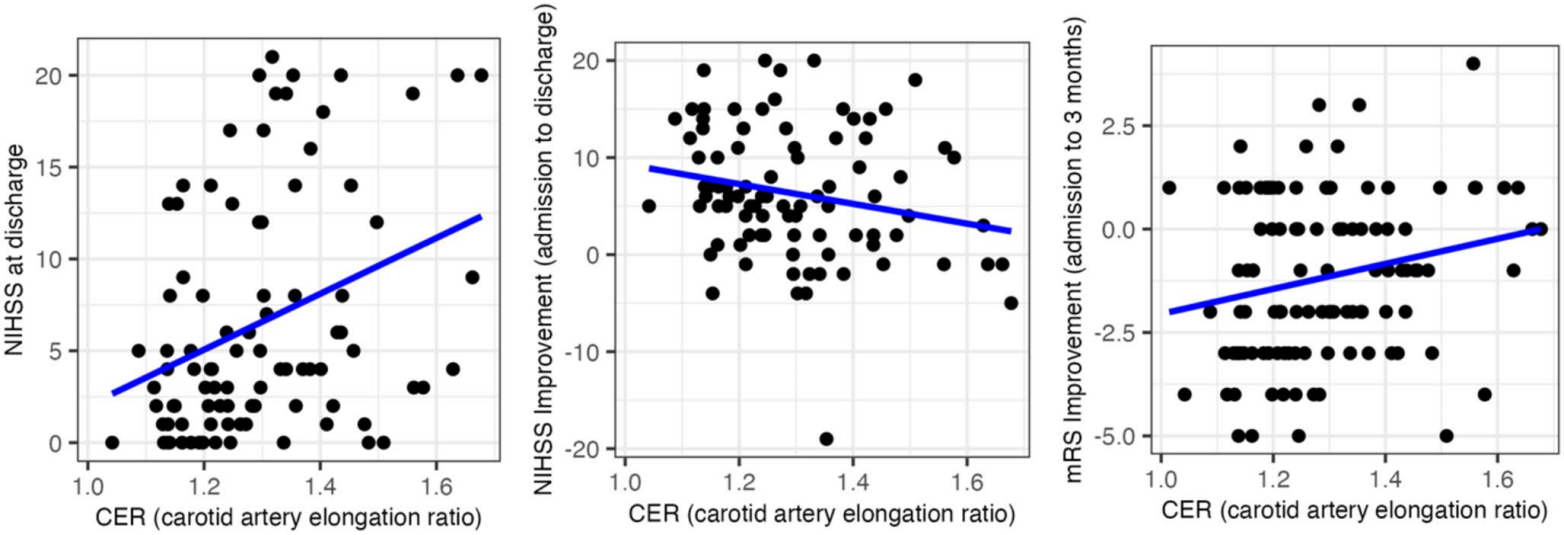
Correlation of Carotid Elongation Ratio with Outcome Parameters. Correlation of NIHSS (National Institutes of Health Stroke Scale) at discharge, Improvement of NIHSS score from admission to discharge and improvement of mRS (modified Rankin Scale) from admission to 3 months as a function of the CER (Carotid Elongation Ratio)

Overall, the median modified Rankin scale (mRS) scores were 5 (IQR 4–5) at admission, 4 (IQR 2–5) at discharge, and 4 (IQR 1–6) at 3 month follow up. 50 patients (42.4%) achieved favorable functional outcome (mRS 0–2). Median NIHSS scores were 14 at admission (IQR 8–17) and 4 at discharge (IQR 2-11.25). There was no significant correlation between CER and NIHSS score at admission (*p* = 0.48, rho = 0.07). A statistically significant moderate correlation between CER and NIHSS score at discharge was found (*p* = 0.001, rho = 0.3). The improvement in NIHSS score from admission to discharge also correlated weakly with CER (*p* = 0.023, rho=-0.2). CER correlated weakly with improvement in mRS from admission to 3 months post-intervention (*p* = 0.031, rho = 0.2) (see Fig. 4).

**Fig. 5:**
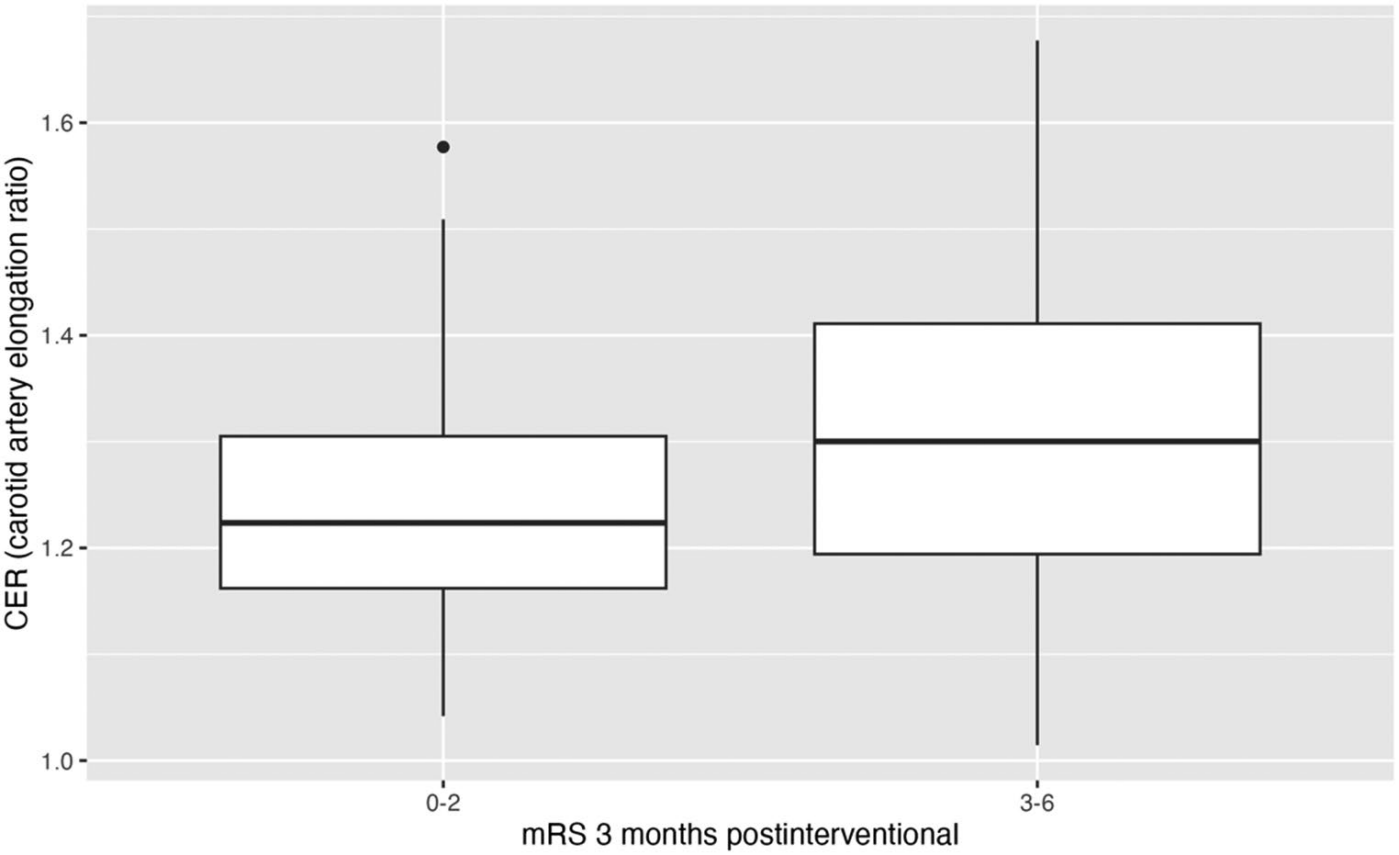
Distribution of 3-month mRS (modified Rankin Scale) in relation to the CER (Carotid Elongation Ratio)

There was no significant correlation of CER with mRS at discharge (*p* = 0.25) or at 3 months post-intervention (*p* = 0.22). However, CER exhibited a statistically significant correlation with the dichotomized 3-month mRS in biserial correlation (cor = 0.2, *p* = 0.03). Furthermore, when adjusting for the influence of relevant comorbidities (diabetes, hypertension, smoking behavior), age and gender, there was a significant partial correlation of CER with dichotomized outcome (cor = 0.2, *p* = 0.048). A statistically significant group difference in CER was found between patients with favorable functional outcome (mRS 0–2; mean CER = 1.2 +-0.1) and unfavorable outcome (mRS 3–6; mean CER 1.3+-0.2) at 3 months post-intervention (*p* = 0.012; Fig. 5).

The results of a multivariate binary logistic regression analysis for prediction of dichotomized mRS outcome after 3 months are shown in Table 3. The model showed a moderate goodness of fit with a *Nagelkerke’s* R^2^ of 0.42. NIHSS score at admission, presence of diabetes, and hypertension as well as intervention time were significant factors. CER showed the highest log Odd’s value (log Odd’s 2.97), suggesting a relatively stronger effect on the probability of the outcome compared to others. However, it did not reach statistical significance, likely due to a large standard error. Analysis of the Variance Inflation Factor (VIF) indicated no relevant collinearity between included factors.

**Table 3:** Binary logistic regression analysis for prediction of dichotomized outcome

Coefficients	log odds	SD	z value	*p* value	VIF [95% CI]
Sex	-0.61	0.59	-1.04	0.298	1.27 [1.10, 1.72]
Age	-0.01	0.03	-0.46	0.643	1.48 [1.24, 1.96]
CER	2.97	2.15	1.38	0.168	1.18 [1.05, 1.66]
Comorbidities					
diabetes	1.83	0.64	2.84	0.004	1.39 [1.18, 1.85]
arterial hypertension	-1.62	0.73	-2.20	0.028	1.63 [1.34, 2.17]
atrial fibrillation	0.75	0.56	1.33	0.184	1.21 [1.07, 1.67]
pAVD	0.85	1.26	0.68	0.497	1.12 [1.02, 1.70]
cigarette smoking	0.75	0.77	0.97	0.334	1.21 [1.07, 1.67]
NIHSS at Admission	0.23	0.06	3.65	< 0.001	1.52 [1.26, 2.02]
mTICI	-0.12	0.28	-0.42	0.671	1.06 [1.00, 2.41]
Groin Puncture to recanalization time	0.03	0.01	1.99	0.047	1.39 [1.18, 1.86]

CER carotid elongation ratio. SD standard deviation. VIF variance inflation factor. pAVD peripheral arterial vascular disease. mTICI modified Thrombolysis in cerebral infarction scale. NIHSS National Institute of Health Stroke Scale

Hemorrhagic transformations and bleedings occurred in 41 cases, accounting for 35% of the total cohort. The distribution was as follows: 34% scattered small petechiae (*n* = 14, class 1a, HI1), 2% confluent petechiae (*n* = 1, class 1b, HI2), 24% subarachnoid hemorrhage (*n* = 10, class 3c), 2% subdural hematoma (*n* = 1, class 3d) and 20% combined subarachnoid hemorrhage and intracerebral hemorrhage (*n* = 8). Furthermore 5% cases of swelling occurred (*n* = 2), 7% of groin bleeding (*n* = 3), 2% of femoral vessel occlusion (*n* = 1) and 5% of arterial dissection (*n* = 2). There was no correlation with CER neither in the presence of complications (rho = 0.06, *p* = 0.46) nor the individual types of complications (rho = 0.07, *p* = 0.46). There was no correlation between the occurrence of complications and the mRS outcome after 3 months (rho = 0.08, *p* = 0.41). Additionally, a group comparison of mean CER values between cases with and without complications showed no statistically significant difference (*p* = 0.57).

## Discussion

The current study aimed to evaluate the influence of carotid artery elongation on the duration of mechanical thrombectomy interventions and patient outcomes in cases of acute middle cerebral artery occlusions. Lacking a gold standard for quantifying Carotid elongation, we have presented an approach for assessing Carotid elongation through an easy-to-assess imaging marker, i.e., CER, which effectively quantifies vessel tortuosity. Moderate or weak correlations of CER were observed with NIHSS scores at discharge as well as with NIHSS improvement from admission to discharge and mRS improvement from admission to 3 months post-intervention. Patients with favorable functional outcomes (mRS 0–2) showed lower CER values, and there was a significant correlation of CER with the dichotomized outcome, also after adjusting for age, gender, and significant cardiovascular risk factors. Furthermore, a weak positive correlation was found between CER and recanalization time, although this correlation was mainly driven by an outlier in our analysis. However, CER showed no significant association with recanalization success.

The definition of carotid elongation lacks standardization in the current literature, leading to various approaches to quantify carotid tortuosity with differences in mathematical structure and practicality of assessment. Some authors prefer image characteristics rather than a ratio to categorize cohorts as “tortuous” or “non-tortuous” or different levels of tortuosity [8, 21]. However, such categorization is quite subjective and prone to immense interrater variability. In the current study, we propose an easy to assess quantitative imaging marker that can be ascertained fast and reliably. Comparable to previous studies from Venturini et al. or Zhang et al., the true length of the vessel was assessed using a centerline measurement in a multiplanar reconstruction with a specialized vessel analysis software [22, 23]. While in these studies the shortest line between the start and end point of the vessel was used to calculate the neck length for the ratio, we observed that this method can be strongly influenced by the neck inclination and positioning of the patient in the CT scanner, which affects reliability and reproducibility of measurements. To address this, we defined the Z-axis parallel to the CT table, which is easier and more reliable to assess. The goal was to enable interventionalists to simplify quantification of the severity of anatomical complexity they might encounter using our ratio in individual cases. Other authors have adopted an approach considering the branching angles of the carotid artery segments. They demonstrated that supra-aortic vessel tortuosity significantly impacts access time in mechanical thrombectomy for the internal carotid artery [24]. Previous studies, for example by Gomez-Pas et al., revealed a correlation between lower carotid tortuosity and faster recanalization [25]. In line with these findings, our study also indicates a mild correlation between the Carotid Elongation Ratio (CER) and recanalization time. However, it is important to emphasize the limitation in our study that this correlation is primarily driven by the presence of an outlier. Therefore, it is conceivable that there is a slight to moderate effect of a complex thrombectomy access path due to elongated carotid arteries on intervention time, but its impact might be mitigated by other factors, for example increasingly flexible neurointerventional material and continuous advancements in interventional techniques. However, other factors possibly prolonging recanalization time must also be considered, e.g. clot composition or challenges in establishing femoral access.

According to Leker et al., carotid tortuosity may have a negative influence on successful recanalization [21]. In contrast, other authors could identify only minor impact of vessel tortuosity on reperfusion success in endovascular thrombectomy [26]. Likewise, in our study, no significant correlation between vessel tortuosity quantified by CER and recanalization success on the mTICI scale was found. This divergence in findings of different studies could be attributed to variations in the definition of tortuosity, differences in interventionalists’ experience, or variations in the materials used in the individual cases of mechanical thrombectomy.

Additionally, beyond investigating its influence on intervention time, we focused on examining the influence of carotid elongation on clinical outcomes. Here, our findings confirm that carotid elongation could indeed impact patient’s outcome. The correlation between CER and the improvement in NIHSS from admission to discharge suggests an effect on patient outcomes. Additionally, our study demonstrated a weak correlation between CER and the improvement in mRS from admission to 3 months post-intervention, indicating a sustained impact on outcomes. Although no significant correlation was found with mRS at discharge or 3 months post-intervention, a statistically significant group difference of CER between patients with favorable outcome and unfavorable outcome at 3 months post-intervention was observed as well as significant correlations with dichotomized outcome. Considering the correlation of carotid elongation, measured as CER, with intervention duration in terms of recanalization time and its impact on patient outcomes, it is important to emphasize that in our study, the correlation with intervention time primarily exists due to the presence of an outlier. However, there appears to be a notable correlation between carotid elongation and poorer outcomes for NIHSS and mRS outcome measures, with clear group differences evident. This leads to the consideration that there may not necessarily be a direct causal chain linking carotid elongation to longer intervention time and subsequently poorer outcomes. The absence of correlation with the recanalization result (mTICI score) supports this notion. Instead, it is possible that carotid elongation could be a risk factor for poor outcomes rather independent of recanalization success or intervention duration. One potential explanation might involve its association with a severe neurovascular risk profile, including arterial hypertension, which could affect overall outcomes and post-stroke recovery [27]. However, (partial) correlation of CER with dichotomized outcome still persisted after controlling for the cardiovascular risk factors diabetes, hypertension and smoking in our study. Other authors have reported different findings, indicating that vessel tortuosity has no significant impact on the clinical outcome [8]. Another study found that there is no difference in the favorable outcome between groups with and without elongated vessels [21]. However, different results regarding clinical improvement in other studies may be attributed to the multifactorial nature of clinical outcome. The impact of carotid elongation on the clinical outcome, particularly on the sustained outcome measured by mRS after 3 months, may be influenced by other factors, such as neurological co-treatments, rehabilitation, and other external variables. There was no association detected between CER and the occurrence of complications. Nevertheless, our study demonstrated a significant impact of carotid elongation on the duration of intervention and patient outcomes. The suggested carotid length/neck ratio serves as an easy-to-assess imaging marker for quantifying vessel tortuosity. Further research will be essential to gain a comprehensive understanding of the significance of the established marker in the context of stroke treatment.

In this study, carotid elongation was discussed as a potential individual risk factor for poor stroke outcomes. It is noteworthy that the current literature provides limited comparable findings in this regard. Furthermore, while a connection between carotid elongation and dissections or aneurysms has been described [22, 23, 28], such a relationship has not been established in the context of stroke outcomes. As the findings from studies regarding carotid artery dissections cannot be directly extrapolated to the interventional stroke care domain, further research is necessary. However, the specific impact of carotid tortuosity on technical success and patient outcomes during stroke treatment involving cerebral artery occlusions remains incompletely understood.

## Limitations

Limitations include the single-center design and the potential for selection bias due to possible exclusion of patients with significantly elongated, non-passable access routes of the carotids who may have presented a particularly poor condition in course of their stroke. The various definitions of carotid elongation in the current literature leads to challenging comparability. No comparison with other calculation methods was performed in this study. Additionally, the proposed imaging marker for carotid elongation was evaluated specifically within the context of one software, i.e., the Sectra IDS7 vessel analysis tool, and its generalizability to measurements taken with other tools remains uncertain. However, due to the straightforward definition of the ratio using the z-axis of the CT scan and center-line determination of the carotid artery, we would expect a good reproducibility with other tools designed for such measurements. Variations in multifactorial causes, such as differing experience levels among interventionalists or other conditions of the patients, could have influenced the outcomes. Details of the individual materials used were not systematically recorded during data collection, which could potentially affect the technical success of the interventions. Nevertheless, interventional procedures followed a standard scheme at the local institution, which should assure a rather low variability of applied materials and practices. Generally, outcome is influenced by multiple factors in the context of stroke treatment, which could potentially introduce bias. Additional studies are required to attain a more profound understanding of the significance of carotid elongation in the context of stroke.

## Conclusion

In summary, based on our findings, carotid elongation ratio (CER) is easily assessable and shows potential as an imaging marker for assessing carotid artery elongation. Carotid elongation had no relevant effect on endovascular treatment and should therefore not guide treatment decisions in the acute phase of stroke. Correlations between CER and clinical improvement after endovascular stroke treatment indicates a potential association between carotid elongation and patient outcomes, which suggests that carotid elongation might be a risk factor for poorer outcome, possibly associated with an increased neurovascular risk profile. However, further research and multi-center evaluation are necessary to validate our approach and should consider carotid elongation as an individual neurovascular risk factor, independent of hypertension or other common neurovascular risk factors.

## Abbreviations

CER: Carotid Elongation Ratio
CTA: Computed Tomography Angiography
F: French
Fig.: Figure
IQR: interquartile range
MRI: Magnetic Resonance Imaging
mRS: modified Rankin scale
NIHSS: National Institute of Health Stroke Scale
PACS: Picture Archiving and Communication System
pAVD: peripheral arterial vascular disease
mTICI: modified Thrombolysis In Cerebral Infarction
VIF: Variance Inflation Factor

## Declarations

### Statistics and biometry

One of the authors has significant statistical expertise.

### Methodology

Retrospective, cross-sectional study, performed at one institution.
